# Effects of low fat diet on inflammatory parameters in individuals with obesity/overweight and non-alcoholic fatty liver disease: A cross-sectional study

**DOI:** 10.1097/MD.0000000000037716

**Published:** 2024-04-12

**Authors:** Nur Bengü Erdem, Evrim Kahramanoğlu Aksoy, Derya Dikmen, Kübra Uçar Baş, Aslihan Ağaçdiken, Merve İlhan Esgin, Zeynep Göktaş

**Affiliations:** a Department of Nutrition and Dietetics, Faculty of Health Sciences, Hacettepe University, Ankara, Turkey; b Department of Gastroenterology, University of Health Sciences, Keçiören Training and Research Hospital, Ankara, Turkey.

**Keywords:** Diet, inflammation, low fat, obesity, weight loss

## Abstract

Nonalcoholic fatty liver disease (NAFLD) is considered one of the most important causes of chronic liver disorders in the world. Dietary pattern is a modifiable risk factor that represents the main target for the prevention and treatment of NAFLD. The aim of this cross-sectional study was to assess the impact of low-fat diet on anthropometric measurements, biochemical, and inflammatory parameters in individuals with obesity/overweight and NAFLD. A total of 108 individuals (*n* = 59 males and *n* = 49 females) aged between 19 and 65 years participated in the 12-week weight loss program. Dietary treatment plans including low-fat diets were randomly prescribed for each individual. Anthropometric measurements were collected by a trained dietitian at baseline and 12-week follow-up. Blood samples were collected for each individual at baseline and 3rd month for biochemical measurements and enzyme-linked immunosorbent assay analysis for tumor necrosis factor-α (TNF-α), interleukin (IL)-6, fibroblast growth factor-21 (FGF-21), chemerin, and irisin levels in plasma. At the end of the study, body weight, body mass index, body fat % body fat mass (kg) reduced significantly in females and males (*P* < .05). Moreover, reductions in waist, hip, and neck circumferences were significant in both groups. Changes in alanine aminotransferase and aspartate aminotransferase levels were significant in 3rd month. After 3 months, reductions in TNF-α, IL-6, and FGF-21 levels were significant in individuals with obesity/overweight and NAFLD. While no significant change in chemerin and irisin levels was found. These results show that low-fat diet over a 12-week period led to improvements in both anthropometric measurements and biochemical parameters in individuals with obesity/overweight and NAFLD.

## 1. Introduction

Nonalcoholic fatty liver disease (NAFLD) is considered one of the most important causes of chronic liver disorders globally. It encompasses a wide range of disorders, including steatosis to nonalcoholic steatohepatitis (NASH), fibrosis, cirrhosis, and hepatocellular cancer.^[1]^ The prevalence of NAFLD is nearly 25% in worldwide, and affecting 1 billion people in the world.^[2]^ Pathophysiological changes such as increased fat content in adipose tissue or skeletal muscles are recognized contributors to the development of NAFLD.^[3]^ Adipose tissue and skeletal muscles are considered endocrine organs because they secrete adipokines and myokines. These molecules can play a role in obesity-related inflammation and NAFLD.^[4]^ In the case of over-expansion of adipose tissue, macrophage infiltration occurs and pro-inflammatory cytokines including interleukin (IL)-6 and tumor necrosis factor-α (TNF-α) and chemerin levels increase.^[5]^ Chemerin is secreted mainly from adipose tissue, the liver, and the lungs. Chemerin levels seem to be higher in people with NAFLD than in healthy control subjects. Chemerin causes glucose intolerance and adipose tissue inflammation in obesity by stimulating immune response and increasing fat accumulation.^[4,6]^ IL-6 is a cytokine that shows both pro-inflammatory and anti-inflammatory properties in NAFLD. TNF-α is associated with insulin regulation related to onset and progression of NAFLD.^[7]^ Fibroblast growth factor-21 (FGF-21) and irisin are myokines playing important roles in NAFLD pathogenesis.^[8]^ Irisin is a myokine that increases energy expenditure, ameliorates insulin sensitivity, and stimulates weight loss. It has been proposed that irisin acts in the development of NAFLD.^[9]^ FGF-21 is a protein primarily secreted from the liver, which maintains energy homeostasis, ameliorates insulin sensitivity, glycolipid metabolism, and reverses hepatic steatosis.^[10,11]^

There is no proven pharmacological therapy for NAFLD. The primary treatment for NAFLD is lifestyle intervention which includes diet and exercise.^[12]^ Dietary patterns are considered modifiable risk factors and represent the main targets for the prevention and treatment of NAFLD. Macro-nutrient changes affect NAFLD treatment as well as energy restriction.^[13]^ Dietary fatty acids are an important factor in the development and progression of NAFLD; high fat diets can be harmful to the liver.^[14]^ Furthermore; inflammatory markers are affected by dietary fat intake which is one of the modifiable risk factors.^[13,15]^ A systematic review assessing randomized controlled trials found that low-fat diet appears to be more successful in reducing in transaminase levels in NAFLD.^[16]^ The recommended dietary intake of fatty acids for adults should be 20% to 35% of daily energy intake.^[13]^ However, this ratio is approximately 40% of daily energy intake on Western-type diets. In literature studies assessing the effect of low-fat diet on inflammatory parameters in individuals with NAFLD is scarce. Furthermore, there is no study evaluating the effect of low-fat diet on chemerin levels in individuals with NAFLD. This study aims to investigate the effects of low-fat diet on liver enzymes and serum levels of inflammatory cytokines such as chemerin, IL-6, TNF-α, and myokines such as irisin, FGF-21 in individuals with overweight, obesity and NAFLD. We hypothesized that low-fat diet results in reductions in anthropometric measurements, biochemical and inflammatory parameters in individuals with obesity/overweight and NAFLD.

## 2. Materials and methods

### 2.1. Study of plan

A total of 108 individuals (*n* = 59 males and *n* = 49 females) aged between 19 and 65 years participated to the 12-week cross-sectional study. Individuals diagnosed with NAFLD via ultrasound by physicians at Gastroenterology Clinic in Keçiören Training and Research Hospital were included in this study in Ankara, between April 2016 and February 2017. Exclusion criteria encompassed individuals with autoimmune diseases, hepatic viruses, mental illnesses, alcohol consumption exceeding 20 g/day, a history of abdominal or bariatric surgery, and pregnancy. This study was approved by the Hacettepe University Non-Interventional Institutional Research Ethics Board and has been performed in accordance with the ethical standards laid down in the 1964 Declaration of Helsinki and its later amendments. All individuals received comprehensive information and signed a consent form before participating in the study. This study was conducted in compliance with The Strengthening the Reporting of Observational Studies in Epidemiology (STROBE) statement. Over the course of this 3-month study, all participants visited the Hacettepe University Department of Nutrition and Dietetics Anthropometry Laboratory twice: once at baseline and again at the 3rd month for anthropometric measurements.

### 2.2. Data collection

A 4-section questionnaire was administered to all individuals in a face-to-face manner by a trained dietitian. The 1st section comprised demographic data, such as age, occupation, and educational status. The 2nd section questioned nutritional habits and physical activity status. The 3rd section collected information regarding smoking and alcohol consumption. Lastly, the 4th section included a 24-hour dietary and physical activity recall.

### 2.3. Anthropometric measurements

All anthropometric measurements were collected by a trained dietitian. Body weight was measured to the nearest 0.1 kg via TANITA TBF-215 (Tokyo, Japan) barefoot without any metal accessorizes. Height was measured with a wall-mounted stadiometer, with participants barefoot and their heads positioned at Frankfurt plane.^[17]^ Body mass index (BMI) was calculated by dividing weight (kg) by height (m) squared.^[17]^ Body composition including total body fat (kg), fat-free mass (kg), and total body water (kg), was measured using TANITA TBF-215. Waist circumference was measured at the midpoint between the lower rib and iliac crest using an inelastic tape measure while participants were standing up.^[18]^ Hip circumference was measured at the maximal part of buttocks. Waist-to-hip ratio (WHR) was calculated by dividing hip circumference (cm) by waist circumference (cm).^[19]^ Neck circumference was measured from laryngeal ledge using an inelastic tape measure while individuals were standing.^[18]^ Abdominal fat was measured while participants were lying down using TANITA VISCAN (Tokyo, Japan). Systolic blood pressure and diastolic blood pressure were measured twice at a moment of rest using OMRON M2 device (Kyoto, Japan) and their mean was recorded as the result.^[20]^

### 2.4. Physical activity

A 24-hour physical activity recall questionnaire was administered to all individuals by a trained dietitian at baseline to determine their PAL. This questionnaire includes 9 sections; sleeping, lying down, sitting, standing with light activities, standing with medium activities, standing with heavy activities, light exercise/sport activities, medium exercise/sport activities, and heavy exercise/sport activities. Individuals were asked about all activities they engaged in during the past 24 hours, with each activity reported in 15 minutes intervals. Activity duration and basal metabolic rate (BMR) values in minutes were multiplied by the physical activity ratio (PAR). The total value was calculated as the total energy expenditure. This calculation is carried through PAR values in FAO/WHO-2001 report.^[21]^ Physical activity levels (PAL) were obtained by dividing the total energy expenditure by the resting metabolic rate. The Harris Benedict equation was used for BMR calculation. Individuals with PAL values between 1.40 and 1.69 as sedentary or light activity lifestyle between 1.70 and 1.99 as active or moderately active lifestyle 2.00 and 2.40 as vigorous or vigorously active lifestyle are classified.

### 2.5. Nutrition treatment

During the baseline visit, following anthropometric measurements, a trained dietitian calculated BMR using Harris Benedict formula.^[22]^ The total dietary energy intake was calculated by taking an individual daily diet energy requirement calories and reducing it by 25% according to gender, physiological situation, and physical activity level for weight loss. The macronutrient content of the diet was set as follows 55% to 60% carbohydrate, 25% lipids, <10% saturated fat, and 10% to 15% protein. The amounts of portions and change lists of the food groups that should be consumed by the individuals were explained. Participants were advised to eliminate desserts, sugared beverages, and snacks. The nutritional treatment was implemented over a period of 12 weeks. Adherence to the diet plan was assessed using 3-day food records (consisting of 1 weekend day and 2 weekdays) every 2 weeks. A photographic food catalog was utilized to determine portion sizes consumed by individuals for the calculation of average energy and nutrient values. The average energy and nutrient values of consumed foods are calculated using a software program (Nutritional Knowledge System BeBiS 7.0 Stuttgart, Germany) by a trained dietitian. These results were compared with recommended dietary allowances (RDA).

### 2.6. Biochemical parameters

Blood samples were collected for each individual after 10 to 12 hour of fasting at baseline and 3rd month. After 20 minutes of centrifuging blood samples were kept at −80 °C. In women participants due to possible adverse effects of sex hormones on blood lipids blood sampling was not collected 1st 5 days of the menstrual cycle. Liver enzymes ALT (alanine aminotransferase), AST (aspartate aminotransferase), and gamma-glutamyl transferase (GGT), fasting plasma glucose (FPG), serum insulin concentrations, Homeostatic Model Assessment for Insulin Resistance (HOMA-IR), Triglycerides, high-density lipoprotein cholesterol (HDL-C) low-density lipoprotein cholesterol (LDL-C), total cholesterol, and creatinine levels were assessed in both groups. HOMA-IR equivalent was used to determine insulin resistance in individuals.^[23]^

HOMA-IR= [(Fasting Blood Glucose mg/dL) * (Fasting Insulin µIU/mL)/405]

### 2.7. Enzyme-linked immunosorbent assay (ELISA)

In blood samples at baseline and 3rd month visit; TNF-α, IL-6 chemerin, irisin, and FGF-21 levels in plasma were measured using commercially available ELISA kits (BOSTER, USA; ELABSCIENCE, Houston, TX) according to manufacturer’s instructions. Plasma concentrations were expressed as pg/mL for TNF-α, IL-6, FGF-21, irisin, and ng/mL for chemerin.

### 2.8. Statistical analyses

Data were evaluated via Statistical Package for the Social Sciences (SPSS). The normality of distribution was evaluated by the Kolmogorov–Smirnov test. *T* tests were applied while comparing the mean values between 2 groups. Post hoc tests were evaluated with LSD if the variations were normal and Games Howell test if not. Correlations between data were calculated by Pearson Correlation test. The data obtained from individuals are presented as mean, standard deviation values. It was considered significant when the *P* value was below .05. Nominal and nonparametric data were evaluated by chi-square test.

## 3. Results

### 3.1. Features of individuals at baseline

A total of 108 individuals with obesity/overweight and NAFLD participated in this 12-week study. The demographic characteristics of the subjects were presented in Table 1. The mean age of the subjects was 42.6 ± 1.2 years. Men had a higher smoking ratio, alcohol consumption, and higher education compared to women (*P* < .05). There was not any significant change between groups in terms of mean age (*P* > .05).

**Table 1:** Demographic characteristics of subjects.

	Females (*n* = 49)	Males (*n* = 59)	*P*	Total (*n* = 108)
Age (years) (mean ± SD)	48.1 ± 10.95	48.1 ± 11.2	.119	42.6 ± 1.17
Smoking (yes, %)	14.3	30.5	.006^a^	23.1
Alcohol consumption (yes, %)	0.0	13.6	.037^a^	7.4
Married (yes, %)	87.8	76.3	.020^a^	98.1
Higher education (yes, %)	40.7	76.3	<.001^a^	30.5

a Differences between groups were evaluated by independent *t* test.

### 3.2. Anthropometric measurements and biochemical parameters of individuals

Anthropometric characteristics and biochemical parameters of the subjects are presented in Tables 2 and 3. BMI, body fat % body fat mass (kg) reduced significantly in both groups (*P* < .05). However, in males, these changes were more significant compared to females (*P* < .001). Fat-free mass (kg) reduction was not significant in both groups. Waist, hip, neck circumferences reduced significantly in both groups. WHR reduction was significant in males. While this reduction was not significant in females. The changes in systolic and diastolic blood pressure were not significant (Table 2). ALT, AST levels reduced significantly compared with baseline (Table 3). Furthermore, triglyceride levels reduced significantly at the end of the study (*P* = .017).

**Table 2:** Anthropometric characteristics and biochemical parameters of the subjects at baseline and 12th week follow-up.

	Females (*n* = 49)		*P*	Males (*n* = 59)		*P*
	Baseline	12th week follow-up		Baseline	12th week follow-up	
Weight (kg)	77.1 ± 3.42	73.5 ± 3.74	.005^a^	85.8 ± 3.36	81.7 ± 3.18	<.001^a^
BMI (kg/m^2^)	31.9 ± 1.22	30.3 ± 1.32	.006^a^	29.0 ± 0.90	27.6 ± 0.80	<.001^a^
Body fat %	40.2 ± 1.58	37.9 ± 1.81	.026^a^	25.8 ± 1.13	23.2 ± 0.89	<.001^a^
Body fat mass (kg)	31.5 ± 2.63	28.6 ± 2.70	.005^a^	22.4 ± 1.67	19.1 ± 1.30	<.001^a^
Fat-free mass (kg)	45.6 ± 0.93	44.8 ± 1.15	.115	63.4 ± 2.10	62.6 ± 2.16	.075
Abdominal fat mass (kg)	14.3 ± 1.05	12.3 ± 1.07	.017^a^	16.3 ± 1.09	14.6 ± 0.98	.002^a^
Waist circumference (cm)	99.7 ± 3.13	96.0 ± 2.98	<.001^a^	101.0 ± 2.05	96.9 ± 1.89	<.001^a^
Hip circumference (cm)	112.7 ± 2.81	109.3 ± 2.78	.001^a^	105.3 ± 1.44	103.2 ± 1.28	<.001^a^
WHR	0.88 ± 0.02	0.87 ± 0.02	.173	0.96 ± 0.01	0.94 ± 0.01	.001^a^
Neck circumference (cm)	35.8 ± 0.42	35.1 ± 0.49	.025^a^	40.4 ± 0.59	39.6 ± 0.64	.016^a^
Systolic blood pressure (mm Hg)	122.5 ± 3.91	121.8 ± 4.81	.841	122.4 ± 3.70	118.6 ± 3.52	.450
Diastolic blood pressure (mm Hg)	77.1 ± 2.25	78.6 ± 2.84	.588	80.7 ± 2.15	80.5 ± 1.89	.903

a *P* values compare baseline and after treatment using paired sample *t* test.

BMI = body mass index, WHR = waist-to-hip ratio.

**Table 3:** Biochemical parameters of the subjects at baseline and 12th week follow-up.

	Females (*n* = 49)		*P*	Males (*n* = 59)		*P*	Total
	Baseline	12th week follow-up		Baseline	12th week follow-up		*P*
Glucose (mg/dL)	115.1 ± 7.73	108.6 ± 5.72	.135	99.6 ± 5.06	101.4 ± 5.77	.667	.383
Insulin (mg/dL)	13.9 ± 3.00	11.8 ± 1.78	.193	11.7 ± 1.35	12.1 ± 2.03	.781	.428
HOMA-IR	3.7 ± 0.84	3.1 ± 0.54	.261	2.9 ± 0.31	3.0 ± 0.46	.856	.473
Creatinine (mg/dL)	0.68 ± 0.02	0.71 ± 0.02	.861	0.90 ± 0.04	0.91 ± 0.05	.792	.393
Total chol (mg/dL)	228.6 ± 23.69	221.5 ± 19.55	.560	221.7 ± 15.99	210.5 ± 15.87	.136	.199
HDL-C (mg/dL)	51.5 ± 3.16	53.4 ± 4.16	.306	44.5 ± 3.10	43.8 ± 2.65	.463	.506
LDL-C(mg/dL)	156.4 ± 18.12	151.8 ± 15.42	.586	153.4 ± 12.17	148.2 ± 11.66	.315	.317
Triglyceride (mg/dL)	217.0 ± 38.36	159.6 ± 17.07	.038^a^	216.1 ± 22.02	196.4 ± 23.29	.265	.017^a^
ALT (U/L)	72.9 ± 10.95	48.0 ± 6.44	.038^a^	107.7 ± 13.36	43.2 ± 5.42	.002^a^	<.001^a^
AST (U/L)	47.7 ± 5.08	36.8 ± 4.58	.032^a^	55.4 ± 6.58	27.3 ± 1.57	.002^a^	<.001^a^
ALP (U/L)	105.1 ± 10.08	100.5 ± 14.02	.420	90.2 ± 8.98	80.1 ± 7.21	.018^a^	.041^a^
Hemoglobin (g/dL)	13.7 ± 0.45	13.7 ± 0.34	.952	15.5 ± 0.43	15.1 ± 0.60	.358	.538

a *P* values compare baseline and after treatment using paired sample *t* test.

ALP = alkaline phosphatase, ALT = alanine aminotransferase, AST = aspartate aminotransferase, HDL-C = high-density lipoprotein cholesterol, LDL-C = low-density lipoprotein cholesterol.

### 3.3. Inflammatory markers and correlations with anthropometric measurements, biochemical parameters

TNF-α, IL-6, and FGF-21 levels reduced significantly compared with baseline. However, reductions in chemerin and irisin levels were not significant at the end of the study (Table 4). We then examined the correlations of anthropometric and biochemical parameters with inflammatory markers. At baseline, BMI and body fat mass were positively correlated with chemerin and TNF-α levels (*P* < .05). At the end of the study, BMI and body fat mass positively correlated with IL-6 and FGF-21 levels. WHR was correlated with irisin levels at baseline and IL-6 at the end of the study (Table 5).

**Table 4:** Inflammatory parameters of the subjects at baseline and 12th week follow-up.

	Females (*n* = 49)		*P*	Males (n = 59)		*P*	Total (*n* = 108)
	Baseline	12th week follow-up		Baseline	12th week follow-up		*P*
Chemerin (ng/mL)	45.4 ± 2.50	45.1 ± 2.12	.861	41.2 ± 3.48	40.5 ± 3.36	.534	.509
TNF-α (pg/mL)	7.9 ± 0.70	6.1 ± 0.53	.038^a^	8.7 ± 0.94	7.7 ± 0.71	.029^a^	.003^a^
IL-6 (pg/mL)	4.01 ± 0.93	3.03 ± 0.44	.027^a^	4.5 ± 0.75	3.3 ± 0.28	.035^a^	.015^a^
FGF-21 (pg/mL)	358.9 ± 85.3	245.5 ± 62.0	.003^a^	267.6 ± 50.2	147.7 ± 25.1	.006^a^	.002^a^
Irisin (pg/mL)	4.5 ± 0.27	4.3 ± 0.27	.572	5.5 ± 0.31	5.2 ± 0.18	.474	.341

a *P* values compare baseline and after treatment using paired sample *t* test.

FGF-21 = fibroblast growth factor-21, IL-6 = interleukin-6, TNF-α = tumor necrosis factor alpha.

**Table 5:** Correlations of anthropometric and biochemical parameters with inflammatory markers at baseline and 12th week follow-up.

	Chemerin		TNF-α		IL-6		FGF-21		Irisin	
	Baseline	Follow-up	Baseline	Follow-up	Baseline	Follow-up	Baseline	Follow-up	Baseline	Follow-up
BMI (kg/m^2^)	*r* = .470	*r* = .274	*r* = .455	*r* = .252	*r* = .024	*r* = .321	*r* = .065	*r* = .435	*r* = .116	*r* = .273
	*P* = .031^a^	*P* = .384	*P* < .001^a^	*P* = .101	*P* = .901	*P* = .038^a^	*P* = .321	*P* = .038^a^	*P* = .839	*P* = .416
Body fat (kg)	*r* = .532^a^	*r* = .377	*r* = .485^a^	*r* = .710^a^	*r* = .053	*r* = .338^a^	*r* = .061	*r* = .425^a^	*r* = .081	*r* = .307
	*P* = .013^a^	*P* = .777	*P* < .001^a^	*P* < .001^a^	*P* = .814	*P* = .028^a^	*P* = .598	*P* = .043^a^	*P* = .985	*P* = .216
WHR	*r* = .075	*r* = .119	*r* = .040	*r* = .275	*r* = .042	*r* = .566^a^	*r* = .006	*r* = .135	*r* = .548^a^	*r* = .122^a^
	*P* = .764	*P* = .153	*P* = .804	*P* = .068	*P* = .596	*P* = .014^a^	*P* = .964	*P* = .298	*P* = .023^a^	*P* = .206^a^
Neck circ. (cm)	*r* = .153	*r* = .030	*r* = .247^a^	*r* = .315	*r* = .040	*r* = .092	*r* = .142	*r* = .059	*r* = .063	*r* = .339
	*P* = .896	*P* = .071	*P* = .020^a^	*P* = .132	*P* = .604	*P* = .379	*P* = .617	*P* = .652	*P* = .664	*P* = .123
ALT (U/L)	*r* = .009	*r* = .026	*r* = −.076	*r* = .475	*r* = −.001	*r* = .212	*r* = −.150	*r* = .200	*r* = .076	*r* = .072
	*P* = .457	*P* = .750	*P* = .330	*P* = .857	*P* = .694	*P* = .627	*P* = .369	*P* = .372	*P* = .683	*P* = .783
AST (U/L)	*r* = .209	*r* = .296	*r* = −.030	*r* = .651^a^	*r* = .019	*r* = .404	*r* = .065	*r* = .276	*r* = .169	*r* = .381
	*P* = .480	*P* = .753	*P* = .278	*P* = .012^a^	*P* = .827	*P* = .375	*P* = .391	*P* = .197	*P* = .433	*P* = .168
HOMA-I*R*	*r* = .210	*r* = .090	*r* = .776^a^	*r* = .788^a^	*r* = .513^a^	*r* = .232	*r* = −.179	*r* = .204	*r* = .220	*r* = .215
	*P* = .892	*P* = .212	*P* = .001^a^	*P* = .001^a^	*P* = .029^a^	*P* = .270	*P* = .872	*P* = .256	*P* = .387	*P* = .249
Total chol (mg/dL)	*r* = −.239	*r* = .171	*r* = .088	*r* = .058	*r* = .032	*r* = .015	*r* = .104	*r* = .145	*r* = .187	*r* = .213
	*P* = .187	*P* = .881	*P* = .565	*P* = .787	*P* = .787	*P* = .379	*P* = .787	*P* = .582	*P* = .425	*P* = .357
T*r*iglycerides (mg/dL)	*r* = −.167	*r* = .349	*r* = .537^a^	*r* = .302	*r* = −.029	*r* = .206	*r* = .116	*r* = .332	*r* = .225	*r* = .159
	*P* = .414	*P* = .204	*P* = .029^a^	*P* = .912	*P* = .664	*P* = .565	*P* = .664	*P* = .098	*P* = .433	*P* = .102

a Correlations were evaluated using Pearson correlation test.

ALT = alanine aminotransferase, AST = aspartate aminotransferase, BMI = body mass index, HOMA-IR = homeostasis model assessment-estimated insulin resistance, WHR = waist-to-hip ratio.

## 4. Discussion

A total of 108 individuals with obesity/overweight and NAFLD participated in this 12-week study. The mean age of the subjects was 42.6 ± 1.2 years. Obesity is considered an important risk factor for patients with NAFLD. Most of the patients are overweight and have high BMI. At baseline, the mean BMI for females and males was 31.9 ± 1.22 kg/m^2^ and 29.0 ± 0.90 kg/m^2^, respectively, in this study. Currently, there is no specific pharmaceutical agent for the treatment of NAFLD, and lifestyle changes aimed at weight loss are considered the cornerstone of treatment. Excess caloric consumption contributes to obesity, which is a leading risk factor for NAFLD and its comorbidities. Macronutrient composition of the diet affects development of NAFLD. Diet therapy involving caloric restriction and changes in dietary macro-nutrient content change play an important role in achieving successful weight loss.^[24]^ Studies implementing hypocaloric diets with a 500 to 750 kcal energy deficit have demonstrated significant weight loss and reductions in BMI among subjects with NAFLD.^[25,26]^ In this study, significant reductions in BMI were observed in both females and males, respectively (*P* = .006, *P* < .001). Weight loss can also have a positive impact on abdominal obesity.^[27]^ In this study, significant reductions in waist circumferences were observed in both females and males (*P* < .001) and changes in abdominal fat mass were significant in both females and males (*P* = .017, *P* = .002).

There is a consensus that gradual weight reduction achieved through caloric restriction, with or without increased physical activity results in improved serum liver enzymes, reduced liver fat, decreased hepatic inflammation, and enhanced liver function.^[27]^ High ALT, AST, and GGT levels are typical features of NAFLD. A systematic review and meta-analysis indicated that weight loss seemed to be associated with significant improvements in biomarkers of liver disease such as ALT, and AST in subjects with NAFLD in the short term.^[28]^ In this study ALT and AST levels reduced significantly at the end of the study (*P* < .001).

NAFLD is associated with IR.^[29]^ In this study, IR was measured using HOMA-IR. There were no significant reductions in HOMA-IR levels at the end of the study (*P* > .05). This could be explained by the short duration of the intervention and the absence of exercise. A systematic review assessing lifestyle changes in patients with NAFLD reported that a combination of diet and exercise is more effective than either of these interventions alone.^[3]^

Diet therapy is a modifiable factor influencing the production of inflammatory markers. A systematic review evaluating dietary interventions on inflammatory markers in patients with NAFLD reported that hypocaloric diets appear to ameliorate the inflammatory profile and dietary fat can affect inflammation by regulating gene expression.^[30]^ Marina et al^[13]^ concluded that a low-fat diet by reducing hepatic triglyceride content can be beneficial for the liver and protect agaisnt NAFLD. It has been reported that dietary fat intake acts as an altering agent that affects inflammatory markers. TNF-α and IL-6 are involved in IR and the progression of NAFLD. In this study, there was a positive correlation between HOMA-IR and TNF-α at baseline (*P* = .001). It was observed that hypocaloric diets with low-fat content significantly reduced TNF-α and IL-6 levels in patients with NAFLD and metabolic syndrome.^[13,31]^ These results are similar to this study. At baseline, there was both positive correlations between body fat mass with TNF-α levels and BMI with IL-6 levels at the end of the study. Significant weight reductions in adipose tissue may have caused reduced levels of these cytokines.

Secretion of chemerin is elevated in individuals with obesity. Body fat mass plays an essential role in determining the quantity of chemerin secretion. Chemerin is an adipokine related to adipogenesis and inflammation.^[32]^ In this study, there was a positive correlation between chemerin, BMI, and body fat at baseline (Table 4). There is a limited number of studies examining the effects of diet therapy on chemerin levels. Studies have demonstrated that chemerin levels decrease concomitantly with weight loss after bariatric surgery.^[6,33]^ A study by Lakhdar^[32]^ showed that chemerin levels were reduced in people with obesity prescribed a hypocaloric diet for 24 weeks. No significant change in chemerin levels was observed at the end of this study. Consequently, achieving more weight loss and implementing a dietary intervention with a longer duration may be more effective in influencing chemerin levels.

Irisin and FGF-21 are myokines involved in energy and glucose metabolism and they have been shown to have positive effects on NAFLD. Irisin levels were stimulated by exercise and converted white adipose tissue into brown adipose tissue.^[34]^ Sajoux et al^[9]^ indicated that irisin levels did not change significantly after hypocaloric with a low-fat diet. Irisin is synthesized during physical activity.^[8]^ It was reported that the impact of diet on irisin levels depends on changes in body composition and fat-free mass^[9]^ In this study there was no significant change in fat-free mass (kg). This result and absence of exercise may have caused non-significant changes in irisin levels.

Circulating FGF-21 levels are elevated in individuals with NAFLD and FGF-21 levels correlated with BMI positively in people with overweight and obesity.^[11]^ Correlations between FGF-21 levels and BMI, waist circumference, and visceral adipose tissues, proposing that obesity is an FGF-21 resistance condition.^[35]^ In this study, FGF-21 levels decreased significantly (*P* = .002). Weight loss in parallel to adipose tissue reduction may have caused a lowering of FGF-21 levels significantly.

There are both strengths and limitations of the current study. In this study, we evaluated the effects of both reduced dietary lipids (25% of dietary energy) and saturated fat (<10% of dietary energy) on anthropometric measurements, biochemical, and inflammatory parameters in individuals with obesity/overweight and NAFLD. Adherence to the diet plan was assessed using 3-day food records (including 1 weekend day and 2 weekdays) in every 2 weeks. In this study, individuals with NAFLD weren’t divided into subgroups such as steatosis or nonalcoholic steatohepatitis. This could potentially result in different cytokine and myokine level outcomes. Another limitation of our study is the absence of exercise as a factor. Especially a combination of diet therapy and exercise might result in significant change on myokine levels. Furthermore, this study is conducted for only 12 weeks. A longer duration might prove more effective in assessing the impact on glucose, lipid metabolism, as well as on and chemerin and irisin levels. This study shows that a low-fat diet over 12 weeks led to improvements in anthropometric measurements and biochemical parameters in individuals with overweight, obesity, and NAFLD. Significant reductions in TNF-α, IL-6, and FGF-21 levels were found while no significant change was observed in chemerin and irisin levels at the end of the study. Further studies are needed to understand the exact mechanisms between diet therapies and inflammatory parameters.

## Acknowledgments

We would like to thank all subjects for their participation in the study.

## Author contributions

**Conceptualization:** Nur Bengü Erdem, Evrim Kahramanoğlu Aksoy, Derya Dikmen, Zeynep Göktaş.

**Investigation:** Nur Bengü Erdem, Kübra Uçar Baş, Aslihan Ağaçdiken, Merve İlhan Esgin.

**Methodology:** Nur Bengü Erdem, Evrim Kahramanoğlu Aksoy, Derya Dikmen, Zeynep Göktaş.

**Writing—original draft:** Nur Bengü Erdem, Zeynep Göktaş.

**Writing—review & editing:** Evrim Kahramanoğlu Aksoy, Derya Dikmen.
